# Management of fibro-osseous lesions of the craniofacial area. Presentation of 19 cases and review of the literature

**DOI:** 10.4317/medoral.18289

**Published:** 2013-03-25

**Authors:** Aldo Suarez-Soto, Mari C. Baquero-Ruiz de la Hermosa, Ignacio Minguez-Martínez, Luis M. Floría-García, Jose Barea-Gámiz, Jose Delhom-Valero, Presentation Risueño-Mata

**Affiliations:** 1 Department of Oral and Maxillofacial Surgery, Hospital Universitario La Fe, Valencia; 2 Head of Service, Department of Oral and Maxillofacial Surgery, Hospital Universitario La Fe, Valencia; 3 Professor of Oral Surgery and Stomatology department and chief of dental school San Pablo CEU, Valencia; 4 Section Head, Department of Oral and Maxillofacial Surgery, Hospital Universitario La Fe, Valencia

## Abstract

Introduction: Fibro-osseous lesions constitute a rare benign type of pathology with a non-odontogenic lineage that affect the craniofacial area. According to Waldrom’s classification, these lesions are divided into: fibrous dysplasia (FD), cemento-ossifying fibroma (COF) and desmoplastic fibroma (DF). Material and Methods: A retrospective study was performed on patients diagnosed with fibro-osseous lesions of the craniofacial area at the Hospital Universitario La Fe, Valencia, during 1987-2009. A total of 19 cases were collected: 15 cases compatible with an FD diagnosis, 3 cases with a COF diagnosis and 1 case with a DF diagnosis. Results: In the differential diagnosis, entities having similar clinical manifestations in the maxillofacial area with possible involvement of teeth or manifestations present as an asymptomatic radiolucent image should be ruled out. We hereby present the management and development of patients treated in our hospital for fibro-osseous lesions. Conclusions: Fibro-osseous lesions share many clinical and radiological characteristics in common, with histological features confirming the nature of the lesion. Management of patients should be individualized and case-specific, assessing the clinical evolution of each case and taking into account the benign nature and growth behavior of this type of tumors.

** Key words:**Fibro-osseous, fibrous dysplasia, cemento-ossifying fibroma, desmoplastic fibroma.

## Introduction

Fibro-osseous lesions constitute a rare type of pathology classified as benign tumors of non-odontogenic lineage that affect the craniofacial area. These lesions were first described by Lichtenstein (1,2) in 1936, and have undergone several changes in terms of classification and identification over the years. According to the classification proposed by Waldrom (3) in 1993, this type of pathology is divided into fibrous dysplasia (FD), cemento-ossifying fibroma (COF) and desmoplastic fibroma (DF).

These entities differ from each other due to different clinical, radiological, and histological characteristics that help make a diagnosis ( Table 1). Surgical treatment of these lesions is favored due to functional or aesthetic reasons derived from the lesion growth. The course of treatment should be planned and individualized in each case based on the manifestations.

**Table 1:**
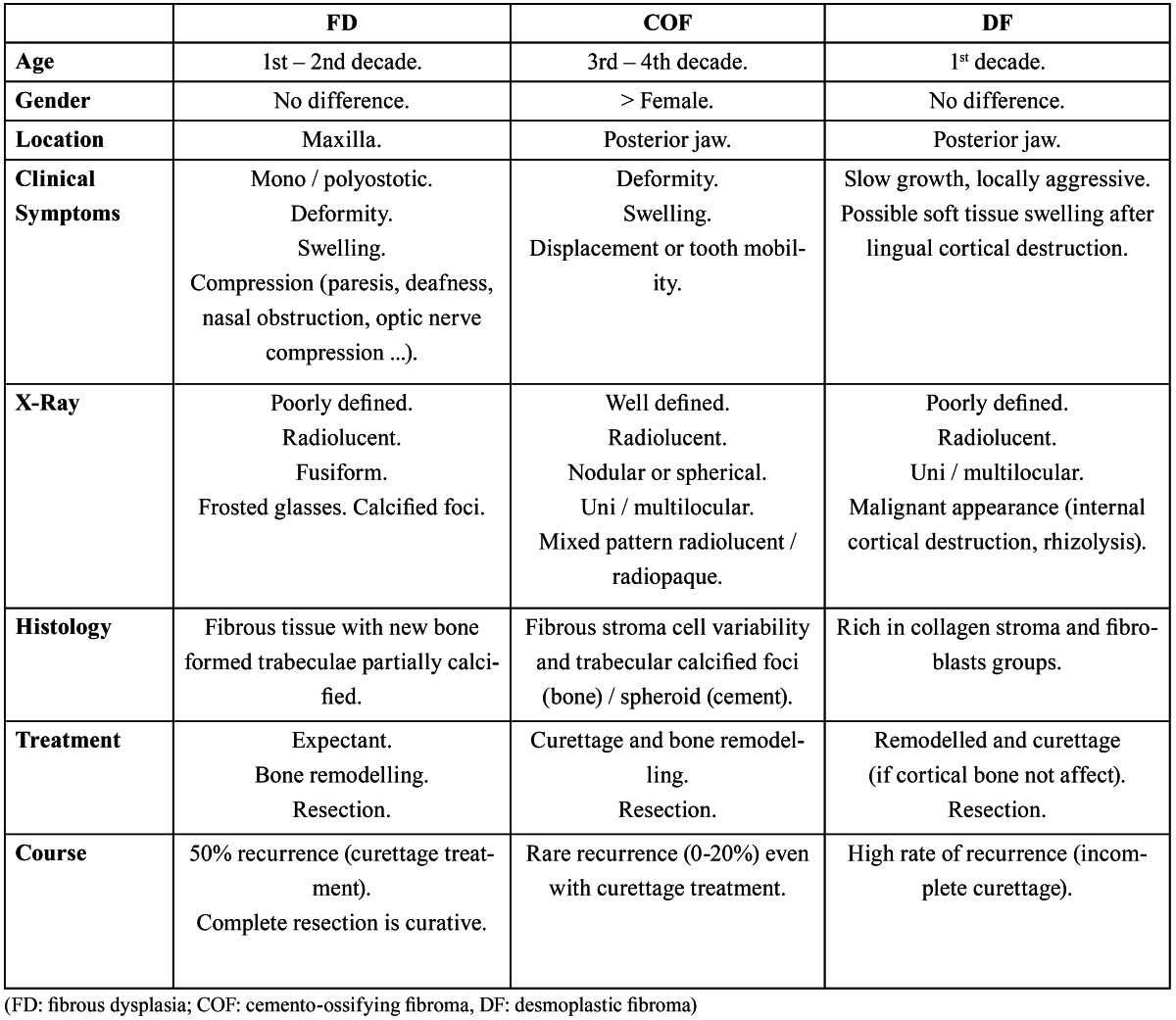
Differential diagnosis between the different entities of fibro-osseous lesions.

In this report, we show our experience in the diagnosis and treatment of this type of lesions. Our objective is to disclose the different types of fibro-osseous lesions that can affect the craniofacial area. We will analyze their clinical, radiological, and histological characteristics and we will try to understand their nature as well as their natural development. Thus, we will try to provide a proper management to patients affected by this type of pathology on an individual basis.

## Material and Methods

A retrospective study was performed on patients diagnosed with fibro-osseous lesions of the craniofacial area at the Hospital Universitario La Fe, Valencia. The study was conducted from 1987 to 2009. The information collected included age, gender, personal history, associated clinical manifestations, accompanying symptoms, radiological and histological information of all cases.

A total of 19 cases were collected. Of the total number of patients, 7 were women and 12 were men. Ages ranged between 4 to 73 years old. 9 pediatric patients and 2 patients aged 65 or more were treated.

The diagnosis was established in 5 cases, as a result of a casual finding during routine X-ray studies, without evidence of clinical manifestations associated with the lesions shown. The patients showed clinical manifestations associated with the lesions in 14 cases. The most common symptom was facial asymmetry, which affected 5 patients. Two patients showed associated facial deformation and local swelling and one of the patients showed facial deformation and pain. In 3 patients, the clinical manifestations included local facial tumors: one patient showed a local tumor and associated dental inclusion, another patient showed dental inclusion associated with local swelling, and the third patient showed only dental inclusions. In 2 of the cases studied, there was a history of previous surgical procedures to treat the same pathology; 2 surgeries in one case and 7 in the other. One of the cases presented a history of post-traumatic osteomyelitis of the mandible that had been treated in 2 previous occasions, which lead to the accidental discovery of FD.

**Figure 1:**
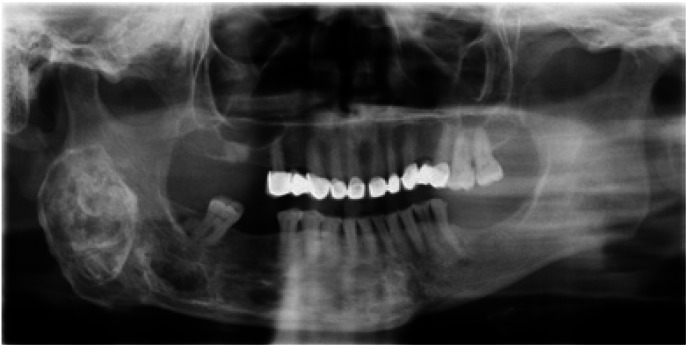
Patient nº15. 26 years evolution mandibular FD. No significant clinical growth, stable and asymptomatic till date.

**Figure 2:**
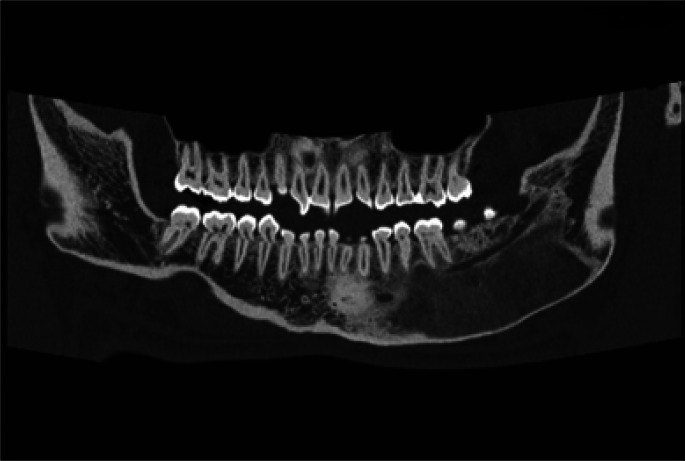
Patient nº 4, mandibular FD diagnosed by an incidental finding in a radiological study, clinically asymptomatic.

**Figure 3:**
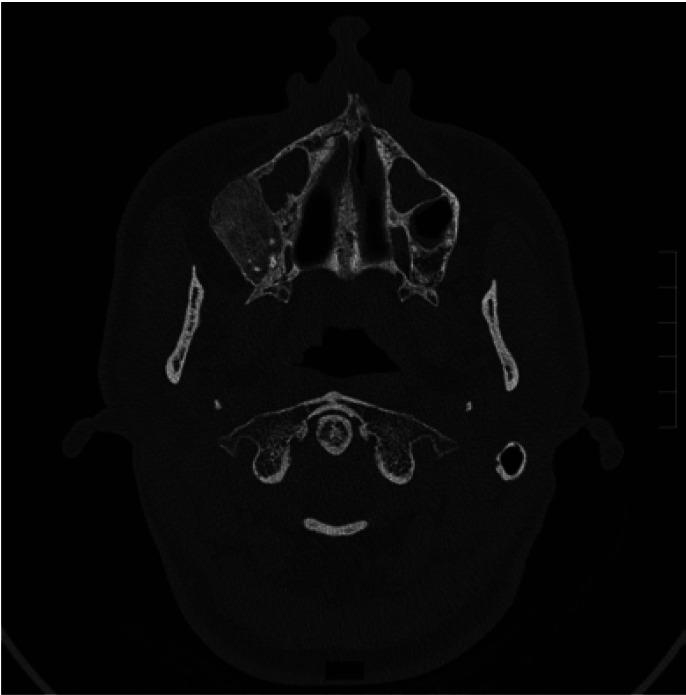
Patient nº 19. Right malar COF lesion that required surgical resection.

Lesions were most frequently found in the mandibular area in 11 of the patients (Figs. 1,2), in the maxillary area in 4 of the patients, in the malar area in 2 of the patients (Fig. 3), with one patient presenting involvement of the parietal area. One of the patients showed conjoint mandibular and maxillary involvement. One case showed a polyostotic involvement which included the mandibular area and the long bones of the upper and lower limbs, with no evidence of endocrine or skin pigmentation alterations.

In all cases, imaging techniques such as orthopantomography (OPG) and computed tomography (CT), were used for the study of lesions. As a complementary test, a bone scan was performed in 5 of the cases, with negative results in each.

An invasive surgery consisting of an incisional biopsy of the lesion was performed in all patients to obtain a histological diagnosis. All procedures were carried out under general anesthesia.

## Results

The management of these clinical cases is summarized in  Table 2. In all cases subjected to surgery, tissue samples were collected and sent for histological assessment. Fifteen cases had a diagnosis compatible with FD, 3 were compatible with a COF diagnosis and 1 case was compatible with a DF diagnosis.

**Table 2:**
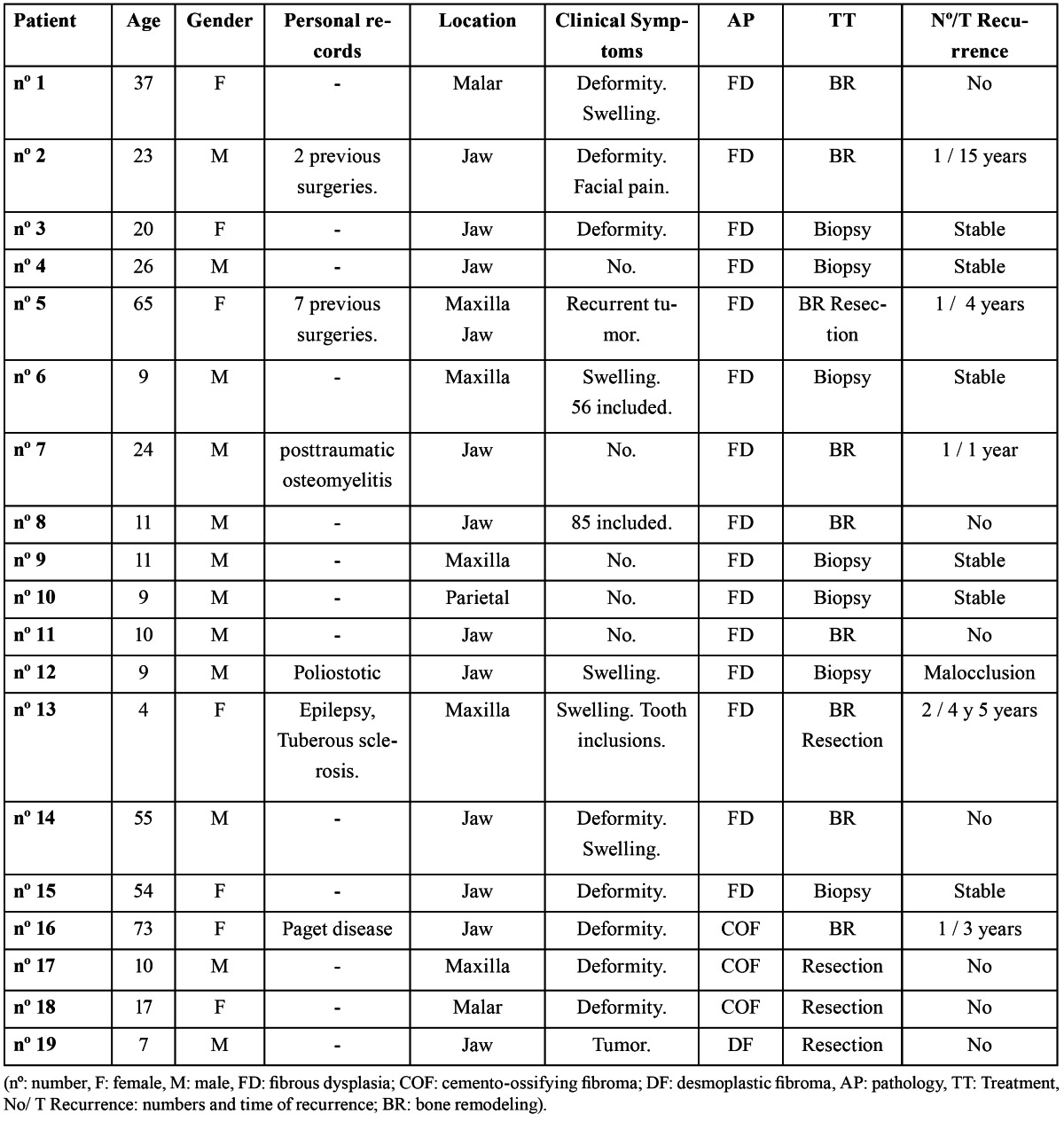
Presentation of the cases.

In 7 of the cases diagnosed with FD, an expectant attitude was maintained. Once the histological confirmation was obtained from the 6 cases diagnosed with FD and from the one diagnosed with COF, a wide bone remodeling was performed on the lesion for aesthetic purposes. Five patients (2 with FD, 2 with COF and 1 with DF) were subjected to a more aggressive surgery, which consisted in the total resection of the lesion. One of the patients with DF needed a mandibular reconstruction with a costochondral graft. In one case of FD, an osteoconductive material was used to regenerate the bone area being treated.

All cases were followed up with clinical controls performed at 1 month, 3 months, and 6 months after the surgery, and an X-ray control was performed six months after the operation.

The patients, who were maintained in an expectant attitude, showed a stable condition during the follow-ups, without evidence of any clinically significant change in the lesions. There was an exceptional case showing growth of the mandibular lesion with clinical repercussion exhibited as occlusal alteration which subsequently required a mandibular bone remodeling.

The post-operative relapse rate in patients with FD was 50%. Of the patients diagnosed with COF, one presented a lesion relapse three years after the surgery, which required a new and larger bone remodeling.

Two cases presented surgery-related complications; one case developed an infection in the treatment area and the other one showed osteonecrosis signs which required an additional procedure involving curettage and bone remodeling.

No malignant transformation from the lesions was observed in any of the cases submitted up to the beginning of the study.

## Discussion

Although there are persistent differences in terms of classification and diagnosis of fibro-osseous lesions in the craniofacial area, there is consensus on the common characteristic of these lesions. All of them show the replacement of normal bone tissue with fibroblast and collagen fiber tissue, with varying quantities of mineralized substances. This investigation is based on the fibro-osseous lesion classification proposed by Waldrom (3) in 1993, who classified them in different entities, namely: fibrous dysplasia (FD), cemento-ossifying fibroma (COF) and desmoplastic fibroma (DF).

FD is considered a benign progressive entity of unknown etiology. It is claimed to have originated as a disruption of the osteogenetic function of osteoblasts that lose their ability to fully differentiate themselves, which impairs bone growth in favor of osteofibrous proliferation. It has been suggested that somatic mutation of the GS-? at osteoblastic cells cause FD in its different forms. It can be clinically manifested in monostotic form with the involvement of a single bone (70%), in polyostotic form with involvement of more than one bone (30%) or in the context of the McCune Albright Syndrome, with associated skin hyperpigmentation and endocrine disorders, such as precocious puberty and/or hyperthyroidism (4). FD affecting multiple adjacent bones in the craniofacial area is considered monostotic. Of the 15 patients studied with FD, only one of them presented polyostotic involvement; associated FD lesions were found in both humeri, and in the left femur and tibia, without skin lesions or endocrine alterations. The entity is mainly manifested in the first and second decades of life, with women being primarily affected, and the jaw being the preferred site. At the time of the diagnosis, 11 of the patients were within this age range, while 4 patients were older. In this study, the rear portion of the jaw was the most frequent site, with men being more affected (2:1). Clinically, it manifests as a painless swelling of the affected bones, and may produce aesthetically visible deformities and symptoms related to vascular or nerve compression such as narrowing of the optic canal. None of the patients showed clinical manifestations related to compression or compromise of structures, the most frequently associated symptoms being asymmetrical facial deformity and site swelling. Typically, lesions alternate periods of quiescence with periods of activity in which the patient develops swelling, discomfort, and increased puffiness; lesions appear as hot spots on the scan, and can simulate osteomyelitis episodes or a low-grade sarcoma. In the latter case, the determination of the cell mutation of the GS-? is useful to confirm the diagnosis of FD (5). Stabilization of the lesions in late puberty or early adulthood is common, although there are some cases reported at a later stage. Even though it is considered a benign entity, there have been cases of sarcomatous degeneration, usually associated with the use of radiotherapy treatments, although there are cases of spontaneous malignant transformation (6). Radiological images may vary depending on the degree of bone present in the lesion. Lesions usually appear as ill-defined, unilocular or multilocular radiolucent lesions with radiopacities on the inside due to the content of bone trabeculae. They may also occur as lesions creating an image of frosted glass. Larger lesions can cause cortical thinning and remodeling, although they can rarely cause a breakdown. Rhizolysis of erupted teeth is a rare finding in this kind of lesions. Histologically, they are characterized by fibrous tissue in which newly formed bone trabeculae, potentially partially calcified, is found. In general, treatment is symptomatic; diagnostic biopsies are often the only procedures needed. In case of clinical manifestations, surgical treatment can range from total resection to curettage of the lesion (7). While most cases achieve a complete cure with the first measure, less aggressive treatments may show up to a 50% of relapses. This type of interventions should preferably be performed during periods of quiescence, since these lesions have a greater vascular component and are more likely to bleed in periods of activity. Six patients of the study were subjected to bone remodeling and 2 were subjected to a complete resection. The post-operative relapse rate in patients with FD was 4/8 (50%.) One patient needed to undergo up to 4 more surgeries. In some patients, the time of lesion relapse ranged from 1 year to 15 years.

COF is considered a benign neoplasm that affects the maxillofacial area, manifesting as an intraosseous or subperiosteal mass. It has unknown etiology and it is typically linked to inflammatory processes, extractions and traumatic backgrounds. These tumors affect mainly the female sex and they usually occur in the third and fourth decades of life. The most common sites are the maxillary teeth areas, primarily the mandibular premolar area (70-80%) and the mandibular ramus. Regarding to the gender of the patients diagnosed with COF, 2 were women and 1 was a man. Two of them were in their second decade of life at the time of the diagnosis, while the other was 73 years old. The site was different in all cases, affecting the left malar area in one case, the rear portion of the mandibular area in another, and the front portion of the upper maxilla in the third case. COF manifests clinically as a slowly growing, painless mass that can result in deformity and facial asymmetry, as it happened in the 3 cases shown. Since it is related to dental roots, it can cause early tooth mobility. Unlike DF, the lesion often appears as a well-defined image in X-rays. In the early stages, the lesion presents itself as radiolucent areas in which bone densities appear as the lesion matures, transforming the image into unilocular or multilocular masses of radiopaque tissue surrounded by a halo of less ossified tissue. Rhizolysis and displacement of affected teeth (8) can be observed. Histologically, lesions present a relatively avascular fibrous stroma consisting of fusiform cells intermingled with bone trabeculae and spheroidal calcifications that resemble cement. Some authors relate the quantity of existing cement with the degree of growth and aggressiveness of the COF (9). While the growth of FD tends to stop at a certain age, COF continues to develop until its resection. Treatment is by means of surgical enucleation of the lesions and curettage of the bone bed. Relapses are rare (0-28%), being more frequent when a simple curettage of the lesion is performed. There is a variant of COF, called active juvenile ossifying fibroma, which affects patients aged 5-15 and has a more aggressive behavior with a tendency to erode surrounding bones. Despite its more aggressive behavior, more mutilating surgeries to treat COF are not necessary (10,11).

DF is a benign, slow-growing and locally aggressive entity. Its etiology is unknown. Hypotheses of its origins include genetic, traumatic and endocrine factors. Any bone can be affected; however, the jaw is most commonly involved within the maxillofacial area (40% preferred site for primary FD), where it may extend to soft tissues due to breakage of the lingual cortex.

It usually appears in the first decade of life, without predilection for either sex.

Clinically, it is manifested by inflammation of the affected area, and can trigger trismus by involvement of the masticatory area. Radiologically, it appears as an ill-defined, unilocular or multilocular radiolucent mass that may cause cortical breaks and root resorption. Histologically, it presents a hypocellular, collagen-rich stroma with groups of fibroblasts inside without any osteoid material. Treatment of this entity involves broad and aggressive en bloc resections of the affected area. Relapses are rare with this type of therapy (12). In reference to the patient diagnosed with DF, once the histological nature of the lesion was known, we decided to perform a complete resection of the lesion located in the rear portion of the left jaw, and rebuilt it with a costochondral graft. The patient has not relapsed up to this date.

Other entities that may manifest clinically as tumors in the maxillofacial area, with or without affectation to the neighboring teeth and appearing as radiolucent images in imaging tests (13-15) should be included in the differential diagnosis of fibro-osseous lesions. Some examples are central giant-cell granuloma, brown tumor of hyperparathyroidism, cherubism, aneurysmal bone cyst, intraosseous vascular malformations, Langerhans cell histiocytosis, non-odontogenic cysts (globulomaxilar cyst, nasolabial cyst, central mandibular cyst, nasopalatine duct cyst) and neurogenic tumors (Schwannoma, neurofibroma and traumatic neuroma). In many cases, these entities can share clinical and radiological characteristics, with histological studies confirming the nature of these lesions.

The different types of fibro-osseous lesions share a common feature, which is the replacement of normal bone tissue by fibrous collagen tissue with variable content of mineral substances. The clinical and radiological characteristics will help with the diagnosis and therapeutic orientation of patients affected by this pathology, with the histological characteristics confirming the nature of the lesion (16,17). Management of patients suffering from fibro-osseous lesions should be individualized and case-specific, assessing the clinical evolution of each case and taking into account the benign nature and growth behavior of these tumors (18-20). According to our experience, we consider it appropriate to maintain an expectant attitude toward FD lesions that do not have significantly clinical or aesthetic impacts. Aggressive surgical procedures should be reserved for relapse cases or cases in which lesion growth compromises adjacent structures.

We consider that, for entities as COF or DF, more aggressive procedures should be planned, namely full enucleations or extensive resections, due to the progressive nature of the tumor in order to prevent relapses and potential further mutilating procedures.

## References

[1] Waldrom CA (1993). Fibro-osseus lesions of the jaws. J Oral Maxillofac Surgery.

[2] PerdigÃo PF, Pimenta FJ, Castro WH, De Marco L, Gomez RS (2004). Investigation of the GSalpha gene in the diagnosis of fibrous dysplasia. Int J Oral Maxillofac Surg.

[3] Kruse A, Pieles U, Riener MO, Zunker Ch, Bredell MG, GrÃtz KW (2009). Craniomaxillofacial fibrous dysplasia: a 10-year database 1996-2006. Br J Oral Maxillofac Surg.

[4] Furin MM, Eisele DW, Carson BS (1997). McCune-Albright syndrome (polyostotic fibrous dysplasia) with intracranial frontoethmoid mucocele. Otolaryngol Head Neck Surg.

[5] Panda NK, Parida PK, Sharma R, Jain A, Bapuraj JR (2007). A clinicoradiologic analysis of symptomatic craniofacial fibro-osseous lesions. Otolaryngol Head Neck Surg.

[6] Feller L, Buskin A, Raubenheimer EJ (2004). Cemento-ossifying fibroma: case report and review of the literature. J Int Acad Periodontol.

[7] Hamner JE, Scofield HH, Cornyn J (1968). Benign fibro-osseous jaw lesions of periodontal membrane origin. An analysis of 249 cases. Cancer.

[8] Dominguete PR, Meyer TN, Alves FA, Bittencourt WS (2008). Juvenile ossifying fibroma of the jaw. Br J Oral Maxillofac Surg.

[9] Schneider M, Zimmermann AC, Depprich RA, KÃbler NR, Engers R, Naujoks CD (2009). Desmoplastic fibroma of the mandible--review of the literature and presentation of a rare case. Head Face Med.

[10] Khanna JN, Andrade NN (1992). Giant ossifying fibroma. Case report on a bimaxillary presentation. Int J Oral Maxillofac Surg.

[11] Farzaneh AH, Pardis PM (2005). Central giant cell granuloma and fibrous dysplasia occurring in the same jaw. Med Oral Patol Oral Cir Bucal.

[12] Vegas Bustamante E, Gargallo Albiol J, Berini AytÃs L, Gay Escoda C (2008). Benign fibro-osseous lesions of the maxillas: analysis of 11 cases. Med Oral Patol Oral Cir Bucal.

[13] Sankaranarayanan S, Srinivas S, Sivakumar P, Sudhakar R, Elangovan S (2011). "Hybrid" lesion of the maxilla. J Oral Maxillofac Pathol.

[14] Sontakke SA, Karjodkar FR, Umarji HR (2011). Computed tomographic features of fibrous dysplasia of maxillofacial region. Imaging Sci Dent.

[15] Yadavalli G (2011). Fibro-osseous Lesions of the Jaw: A Report of Two Cases. J Clin Imaging Sci.

[16] Koury ME, Regezi JA, Perrott DH, Kaban LB (1995). "Atypical" fibro-osseous lesions: diagnostic challenges and treatment concepts. Int J Oral Maxillofac Surg.

[17] Worawongvasu R, Songkampol K (2010). Fibro-osseous lesions of the jaws: an analysis of 122 cases in Thailand. J Oral Pathol Med.

[18] Posnick JC (1998). Fibrous dysplasia of the craniomaxillofacial region: current clinical perspectives. Br J Oral Maxillofac Surg.

[19] Rosenberg A, Mokhtari H, Slootweg PJ (1999). The natural course of an ossifying fibroma. A case report. Int J Oral Maxillofac Surg.

[20] Sakamoto M, Hayashida T, Sugasawa M (2001). A case of fibrous dysplasia of the temporal bone: evaluation of treatment performed 23 years ago. Otolaryngol Head Neck Surg.

